# Oxidised Met^147^ of human serum albumin is a biomarker of oxidative stress, reflecting glycaemic fluctuations and hypoglycaemia in diabetes

**DOI:** 10.1038/s41598-019-57095-2

**Published:** 2020-01-14

**Authors:** Akari Momozono, Yoshio Kodera, Sayaka Sasaki, Yuzuru Nakagawa, Ryo Konno, Masayoshi Shichiri

**Affiliations:** 1 Department of Endocrinology, Diabetes and Metabolism, Kitasato University School of Medicine, 1-15-1 Kitasato, Minami-ku, Sagamihara, Kanagawa 252-0374 Japan; 2 Department of Physics and Kitasato University School of Science, 1-15-1 Kitasato, Minami-ku, Sagamihara, Kanagawa 252-0373 Japan; 3 Center for Disease Proteomics, Kitasato University School of Science, 1-15-1 Kitasato, Minami-ku, Sagamihara, Kanagawa 252-0373 Japan

**Keywords:** Mass spectrometry, Predictive markers, Diabetes

## Abstract

Oxidative stress has been linked to a number of chronic diseases, and this has aroused interest in the identification of clinical biomarkers that can accurately assess its severity. We used liquid chromatography-high resolution mass spectrometry (LC-MS) to show that oxidised and non-oxidised Met residues at position 147 of human serum albumin (Met^147^) can be accurately and reproducibly quantified with stable isotope-labelled peptides. Met^147^ oxidation was significantly higher in patients with diabetes than in controls. Least square multivariate analysis revealed that glycated haemoglobin (HbA_1c_) and glycated albumin (GA) did not significantly influence Met^147^ oxidation, but the GA/HbA_1c_ ratio, which reflects glycaemic excursions, independently affected Met^147^ oxidation status. Continuous glucose monitoring revealed that Met^147^ oxidation strongly correlates with the standard deviation of sensor glucose concentrations and the time spent with hypoglycaemia or hyperglycaemia each day. Thus, glycaemic variability and hypoglycaemia in diabetes may be associated with greater oxidation of Met^147^. Renal function, high-density lipoprotein-cholesterol and serum bilirubin were also associated with the oxidation status of Met^147^. In conclusion, the quantification of oxidised and non-oxidised Met^147^ in serum albumin using our LC-MS methodology could be used to assess the degree of intravascular oxidative stress induced by hypoglycaemia and glycaemic fluctuations in diabetes.

## Introduction

Oxidative stress is involved in a number of disease processes, including cardiovascular diseases^1,2^, diabetes^3–7^, chronic kidney disease^8–10^, cancer^11,12^, hypertension^2^ and neurodegenerative disorders^13,14^. Oxidative stress is also believed to be associated with ageing-associated disorders^15,16^. Functional oxidative modification of biomolecules, including intravascular and cellular proteins, may have a causal role in the cellular dysfunctions that are involved in disease pathophysiology^17,18^. The identification of clinical biomarkers of the severity of exposure to oxidative stress has been the intense focus of many researchers^19,20^, because they could be used to predict the development of major human diseases. Because the quantification of reactive oxygen species is difficult, given their very short half-lives, the measurement of stable by-products generated under conditions of oxidative stress remains a popular approach to the monitoring of free radical-influenced processes^20^.

Methionine (Met), a sulfur-containing amino acid, is an important antioxidant that contributes to the structure and stability of proteins^21^. Met is readily oxidised to form Met sulfoxide (MetO), which can be reduced back to Met by MetO reductases^22–26^. Because of this instability of Met and MetO, their quantification has not been a widely used method for the assessment of the degree of oxidative stress^27^. However, we have recently found that the mass spectral intensity of serum tryptic peptides containing oxidised and non-oxidised Met residues can be very stably and reproducibly measured using liquid chromatography-high resolution mass spectrometry (LC-MS), irrespective of the time the blood sample is left to clot at room temperature before centrifugation or repeated freeze/thaw cycles^28^. This may be because of the absence of MetO reductase activity in human blood^29^, although these have not been well characterised to date.

In the present study, we have quantified the levels of oxidised and non-oxidised Met at position 147 of human serum albumin (Met^147^), to determine whether the oxidation status of this residue reflects the oxidative stress induced during disease pathophysiology, and ultimately whether this might represent a useful biomarker of oxidative stress. To this end, we have improved our mass spectrometric methodology for the accurate and stable quantification of such residues in clinically-derived samples.

## Results

### Improved methodology for the quantification of oxidised and non-oxidised Met-containing serum tryptic peptides

We first sought to improve the accuracy and reproducibility of our previous methodology for the quantification of the oxidation of Met residues in serum tryptic proteins^28^. We previously found that the ratio of trypsin-digested serum albumin fragments containing oxidised and a non-oxidised Met residues at position 147 of human serum albumin, Alb(Met^147^O) and Alb(Met^147^), is one of the most promising potential clinical biomarker of intravascular redox status among the Met-containing tryptic serum proteins identified using a proteomic strategy^28^. Because the use of stable isotope-labelled peptides is known to enable the accurate quantification of peptide concentrations in biological samples, we synthesized two stable isotope-labelled peptides, SI-Alb(Met^147^) and SI-Alb(Met^147^O), corresponding to the tryptic peptides, Alb(Met^147^) and Alb(Met^147^O), respectively.

**Figure 1:**
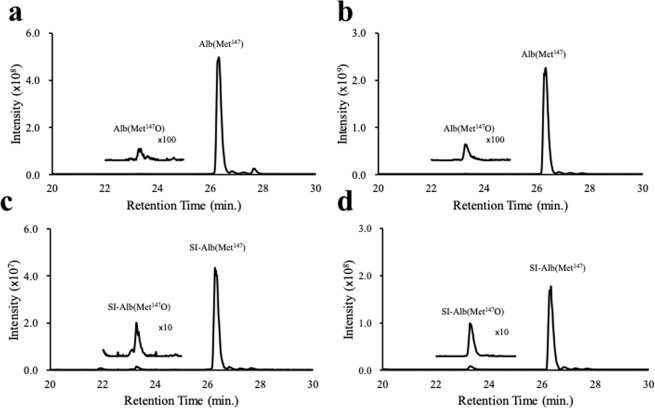
Representative extracted ion chromatograms (XICs) of tryptic peptides containing oxidised and non-oxidised Met^147^ residue and relevant stable isotope-labelled peptides. The stable isotope-labelled peptides, SI-Alb(Met^147^) and SI-Alb(Met^147^O), were spiked to the serum prior to trypsin digestion. XICs with charge states of three (**a**,**c**) and four (**b**,**d**) of endogenous (Alb(Met^147^) and Alb(Met^147^O)) (**a**,**b**) and stable isotope-labelled peptides (**c**,**d**) are presented. Oxidised peptides were magnified 100-fold and 10-fold, respectively, and are shown above the original peaks.

Our previous methodology, which did not use stable isotope-labelled peptides, quantified the signal intensity of Alb(Met^147^O) and Alb(Met^147^), and employed their ratio as an indicator of oxidised Met. In the current analysis, the serially diluted stable isotope-labelled peptides, SI-Alb(Met^147^) and SI-Alb(Met^147^O), were spiked into serum samples from participants prior to tryptic digestion and LC-MS analysis to generate the extracted ion chromatogram (XIC) intensities for the two endogenous tryptic peptides, Alb(Met^147^) and Alb(Met^147^O), and the corresponding stable-isotope-labelled peptides, SI-Alb(Met^147^) and SI-Alb(Met^147^O) (Fig. 1). The serum concentrations of Alb(Met^147^) (${{\rm{C}}}_{{{\rm{Alb}}({\rm{Met}}}^{147})}$) and Alb(Met^147^O) $({{\rm{C}}}_{{\rm{Alb}}({{\rm{Met}}}^{147}{\rm{O}})})$ were then extrapolated from the XICs generated using the respective endogenous peptides and the corresponding spiked stable isotope-labelled peptides. The oxidation ratio for Met^147^ was obtained by dividing ${{\rm{C}}}_{{\rm{Alb}}({{\rm{Met}}}^{147}{\rm{O}})}$ by ${{\rm{C}}}_{{{\rm{Alb}}({\rm{Met}}}^{147})}$. Standard calibration curves were generated using serially diluted serum samples *vs*. quantified Alb(Met^147^) or Alb(Met^147^O), and resulting equations from the regression analyses demonstrated very high degrees of linearity (Supplementary Fig. S1). This updated method, using stable isotope-labelled peptides, yielded a coefficient of variation (%CV) of 9.8%, while the original method, which used signal intensity ratio of Alb(Met^147^O) and Alb(Met^147^), yielded a %CV of 19.7% (n = 17). Therefore, subsequent quantifications were performed using ${{\rm{C}}}_{{\rm{Alb}}({{\rm{Met}}}^{147}{\rm{O}})}$/${{\rm{C}}}_{{{\rm{Alb}}({\rm{Met}}}^{147})}$ values determined using the respective stable isotope-containing peptides.

**Figure 2:**
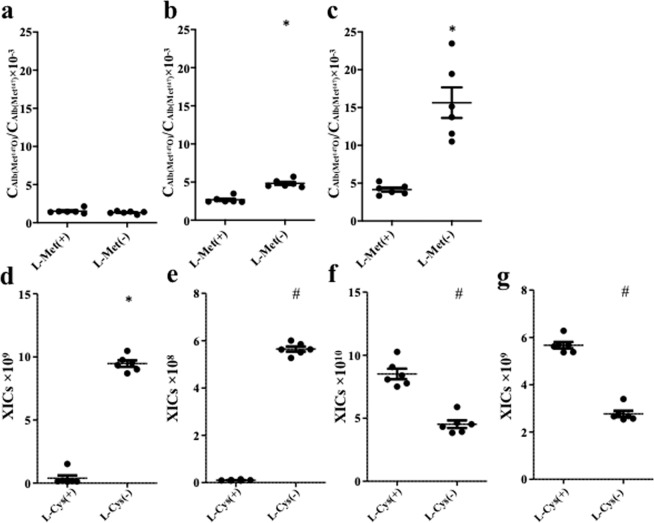
Suppressive effects of L-Met and L-Cys on the spontaneous oxidation of Met and the carbamidomethylation of the N-terminal amino acid of Alb(Met^147^). Serum samples obtained from six healthy participants were digested with trypsin and, after removal of surfactant, stored at −80 °C with (L-Met(+)) or without (L-Met(−)) the addition of excess L-Met for 0 (**a**), 7 (**b**) or 28 (**c**) days before determining ${{\rm{C}}}_{{\rm{Alb}}({{\rm{Met}}}^{147}{\rm{O}})}$/${{\rm{C}}}_{{{\rm{Alb}}({\rm{Met}}}^{147})}$_)_. Trypsin-digested serum samples with or without the addition of excess L-Cys were analysed using LC-MS and the XICs of N-terminally carbamidomethylated Alb(Met^147^) (**d**), SI-Alb(Met^147^) (**e**), uncarbamidomethylated Alb(Met^147^) (**f**) and SI-Alb(Met^147^) (**g**) were determined. Horizontal bars represent the mean ± SEM. *p < 0.05 *vs* L-Met(+) or L-Cys(+), ^#^*p* < 0.005 *vs* L-Met(+) or L-Cys(+).

We next evaluated the benefits of using of L-Met and L-cysteine (L-Cys) to prevent the spontaneous oxidation of Met and the carbamidomethylation of N-terminal amino acid residues. The addition of L-Met after the tryptic digestion of serum samples suppressed the spontaneous oxidation of Alb(Met^147^). ${{\rm{C}}}_{{\rm{Alb}}({{\rm{Met}}}^{147}{\rm{O}})}$/${{\rm{C}}}_{{{\rm{Alb}}({\rm{Met}}}^{147})}$, measured in serum samples from six healthy participants without the addition of L-Met, increased after a week or a month of storage (Fig. 2a–c). In contrast, the addition of excess L-Met to the serum samples markedly suppressed this increase (Fig. 2a–c). We next compared the XICs of Alb(Met^147^) and SI-Alb(Met^147^), with and without the use of L-Cys. The addition of excess L-Cys prior to the trypsin digestion of serum samples inhibited carbamidomethylation at the N-terminus of these peptides (Fig. 2d,e), leading to higher XIC values for uncarbamidomethylated Alb(Met^147^) and SI-Alb(Met^147^) (Fig. 2f,g). Therefore, we added excess L-Cys and L-Met prior to and immediately after the enzymatic digestion of serum samples in subsequent experiments.

The effect of long-term storage on Met oxidation was evaluated using SI-Alb(Met^147^) and SI-Alb(Met^147^O), and excess L-Cys and L-Met. ${{\rm{C}}}_{{\rm{Alb}}({{\rm{Met}}}^{147}{\rm{O}})}$/${{\rm{C}}}_{{{\rm{Alb}}({\rm{Met}}}^{147})}$ did not significantly change after 2 years of storage at −80 °C (0.001267 ± 0.0001391, compared with the value obtained immediately after blood withdrawal: 0.001447 ± 0.0002763; n = 6; *p* = 0.2188). We next determined whether the length of the clotting time prior to serum separation affected the spontaneous oxidation of Alb(Met^147^). Blood samples were allowed to clot at room temperature, and the obtained sera were alkylated and trypsin-digested for subsequent LC-MS analysis. ${{\rm{C}}}_{{\rm{Alb}}({{\rm{Met}}}^{147}{\rm{O}})}$/${{\rm{C}}}_{{{\rm{Alb}}({\rm{Met}}}^{147})}$, determined in samples centrifuged after periods of time on the bench of between 10 min and 6 h, did not show any appreciable differences (0.001331 ± 0.00008874, 10 min; 0.001325 ± 0.00006762, 30 min; 0.001221 ± 0.0001424, 1 h; 0.001264 ± 0.0001149, 2 h; 0.001267 ± 0.0001608, 3 h; 0.001312 ± 0.0001276, 6 h; *p* = 0.5783). Therefore, ${{\rm{C}}}_{{\rm{Alb}}({{\rm{Met}}}^{147}{\rm{O}})}$/${{\rm{C}}}_{{{\rm{Alb}}({\rm{Met}}}^{147})}$ is a robust and reproducible measurement that is not affected by clotting time.

### Oxidised Met ratio in diabetes

**Figure 3:**
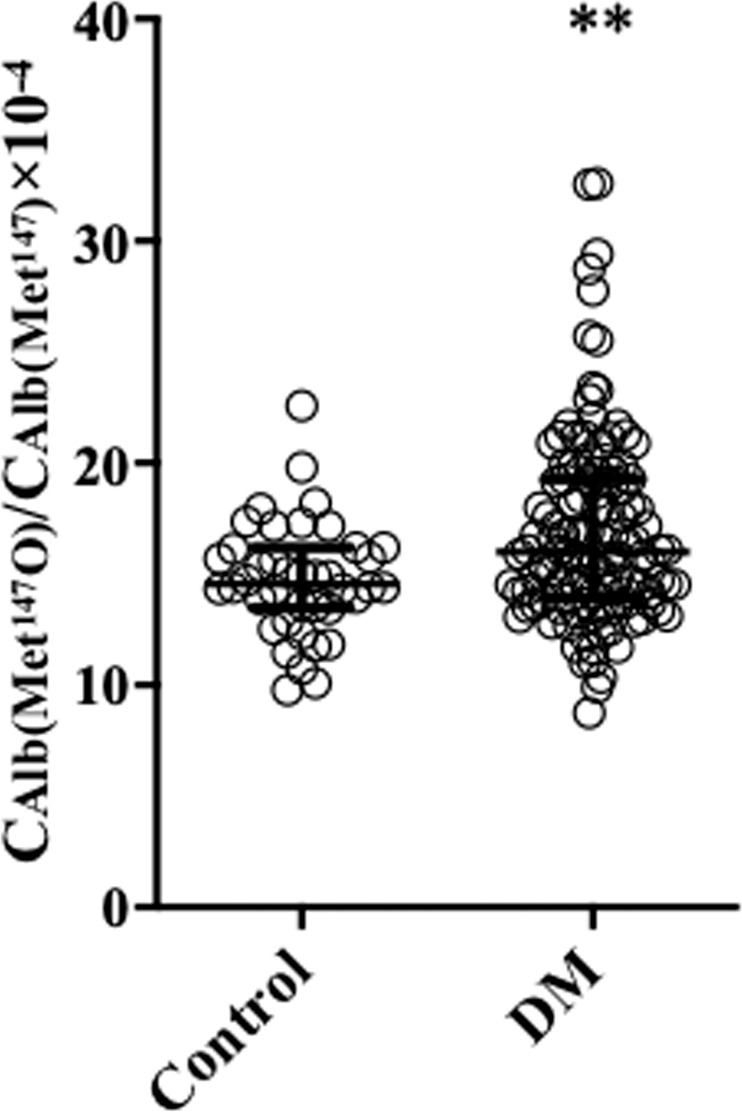
Levels of Met oxidation in diabetic and non-diabetic participants. The serum concentrations of Alb(Met^147^) (${{\rm{C}}}_{{{\rm{Alb}}({\rm{Met}}}^{147})}$) and Alb(Met^147^O) ${{\rm{C}}}_{{{\rm{Alb}}({\rm{Met}}}^{147})}$ were determined using the XICs generated by LC-MS analyses of Alb(Met^147^), Alb(Met^147^O), SI-Alb(Met^147^) and SI-Alb(Met^147^O) peptides. ${{\rm{C}}}_{{\rm{Alb}}({{\rm{Met}}}^{147}{\rm{O}})}$/${{\rm{C}}}_{{{\rm{Alb}}({\rm{Met}}}^{147})}$ was determined in 40 healthy volunteers (Control) and 124 diabetic participants (DM). **p < 0.01 compared with Control.

We next measured ${{\rm{C}}}_{{\rm{Alb}}({{\rm{Met}}}^{147}{\rm{O}})}$/${{\rm{C}}}_{{{\rm{Alb}}({\rm{Met}}}^{147})}$ in 40 healthy volunteers and 124 patients with diabetes (Table 1). ${{\rm{C}}}_{{\rm{Alb}}({{\rm{Met}}}^{147}{\rm{O}})}$/${{\rm{C}}}_{{{\rm{Alb}}({\rm{Met}}}^{147})}$ levels were significantly higher in the diabetic patients than in the healthy volunteers (Fig. 3). Single regression analysis revealed that age, glycated albumin (GA)/glycated haemoglobin (HbA_1c_), blood urea nitrogen (BUN) serum creatinine (Cr) and uric acid positively correlated with ${{\rm{C}}}_{{\rm{Alb}}({{\rm{Met}}}^{147}{\rm{O}})}$/${{\rm{C}}}_{{{\rm{Alb}}({\rm{Met}}}^{147})}$ level, and this negatively correlated with body mass index (BMI), estimated glomerular filtration rate (eGFR) and serum total bilirubin (Table 2). Least square multivariate analysis was undertaken using these statistically significant parameters as explanatory variables, as well as those reported to have an antioxidant activity, and revealed that GA/HbA_1c_, eGFR, high-density lipoprotein (HDL)-cholesterol and total bilirubin significantly and independently influenced ${{\rm{C}}}_{{\rm{Alb}}({{\rm{Met}}}^{147}{\rm{O}})}$/${{\rm{C}}}_{{{\rm{Alb}}({\rm{Met}}}^{147})}$ level (Table 3).

**Table 1:** Characteristics of the enrolled subjects.

	Diabetes	Healthy volunteers	p value
N (male/female)	124 (70/54)	40 (25/15)	
Type2/Type1	94/30		
Age (years)	54.3 ± 13.9	53.2 ± 16.4	0.3363
Body weight (kg)	69.8 ± 21.1	59.4 ± 12.4	0.0409
Body mass index (kg/m^2^)	25.9 ± 6.4	22.5 ± 2.8	0.0498
HbA1c (%)	9.0 ± 2.4	5.5 ± 0.3	<0.0001
eGFR (mL/min/1.73 m^2^)	72.0 ± 24.9	75.2 ± 13.5	0.4338
Total cholesterol (mg/dL)	190.3 ± 43.1	197.6 ± 30.7	0.0547
Triglyceride (mg/dL)	150.1 ± 119.4	92.9 ± 48.1	0.0015
HDL-cholesterol (mg/dL)	55.0 ± 17.8	71.3 ± 17.4	<0.0001
LDL-cholesterol (mg/dL)	108.1 ± 33.5	111.4 ± 23.6	0.1500
Uric acid (mg/dL)	5.5 ± 2.6	4.9 ± 1.0	0.3514
Total bilirubin (mg/dL)	0.7 ± 0.4	0.8 ± 0.5	0.2719

HbA1c = glycated hemoglobin; HDL = high-density lipoprotein-cholesterol; LDL = low-density lipoprotein-cholesterol; eGFR = estimated glomerular filtration rate.

**Table 2:** Correlations between the serum level of methionine oxidation and other parameters (univariate analyses).

Parameters	r	p
Age (years)	0.2253	0.0037
Male: Female		0.3858
BMI	–0.2853	0.0007
HbA1c (%)	–0.0171	0.8413
GA (%)	0.0672	0.4961
GA/HbA1c	0.2946	0.0023
BUN (mg/dL)	0.2899	0.0002
Cr (mg/dL)	0.3708	<0.0001
eGFR (mL/min/1.73 m^2^)	–0.3471	<0.0001
Uric acid (mg/dL)	0.1815	0.0233
Total bilirubin (mg/dL)	–0.1647	0.0426
Triglyceride (mg/dL)	–0.1221	0.1241
HDL-cholesterol (mg/dL)	–0.0449	0.5741
LDL-cholesterol (mg/dL)	–0.1067	0.1809

BMI = body mass index; HbA1c = glycated haemoglobin; GA = glycated albumin; BUN = blood urea nitrogen; Cr = creatinine; eGFR = estimated glomerular filtration rate; HDL = high-density lipoprotein; LDL = low-density lipoprotein.

**Table 3:** Multivariate analysis of the relationship between the serum level of methionine oxidation and other participant characteristics.

Parameters	β	F	p
Age	0.0132	0.0084	0.9273
Male: Female	0.0649	0.4141	0.5224
BMI	–0.1924	1.5425	0.2192
GA/A1c ratio	0.5014	14.2968	0.0004
eGFR (mL/min/1.73 m^2^)	–0.2991	6.1597	0.0159
Uric acid (mg/dL)	0.1572	1.1847	0.2808
Total bilirubin (mg/dL)	–0.2269	5.0440	0.0285
HDL-cholesterol (mg/dL)	–0.2348	4.7193	0.0339
Metformin (+/−)	0.0824	0.5725	0.4523
Statin (+/−)	–0.1417	2.0402	0.1585
ACEI/ARB (+/−)	–0.1382	1.6209	0.2080

BMI = body mass index; HbA1c = glycated hemoglobin; GA = glycated albumin;

eGFR = estimated glomerular filtration rate; HDL = high-density lipoprotein;

ACEI = angiotensin converting enzyme inhibitor; ARB = angiotensin receptor blocker.

**Figure 4:**
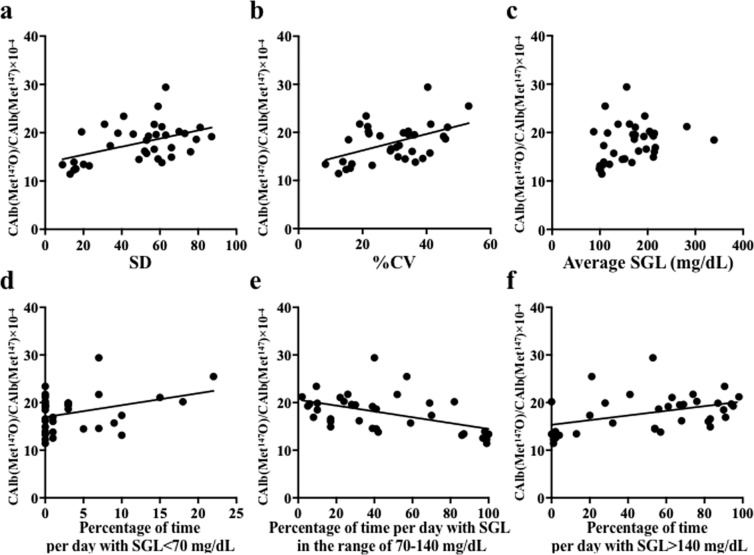
Relationship between Met oxidation and blood glucose profile, evaluated using continuous glucose monitoring. Continuous glucose monitoring was performed in 35 participants for 4–7 days and the sensor glucose levels (SGL) over the entire monitoring period were used to calculate the standard deviation (SD) (**a**), % coefficient of variation (%CV) (**b)** and the mean SGL (**c**) values. The relative lengths of time with SGL < 70 mg/dL (**d**), 70–140 mg/dL (**e**) and >140 mg/dL (**f**) were plotted against ${{\rm{C}}}_{{\rm{Alb}}({{\rm{Met}}}^{147}{\rm{O}})}$/${{\rm{C}}}_{{{\rm{Alb}}({\rm{Met}}}^{147})}$_,_ and the corresponding regression line is shown.

From the study sample of 164 participants, 35 (17 men and 18 women; 28 diabetic and 7 non-diabetic participants; 47.2 ± 15.5 years) had their ${{\rm{C}}}_{{\rm{Alb}}({{\rm{Met}}}^{147}{\rm{O}})}$/${{\rm{C}}}_{{{\rm{Alb}}({\rm{Met}}}^{147})}$ measured while undergoing continuous glucose monitoring (CGM). The standard deviation (SD), %CV and the mean sensor glucose level (SGL) were calculated over 4–7-day monitoring periods. The periods of time during each day the participant was hypoglycaemic (SGL < 70 mg/dl), normoglycaemic (70 mg/dl < SGL < 140 mg/dl) and hyperglycaemic (140 mg/dl < SGL) were also calculated. The SD and %CV significantly correlated with ${{\rm{C}}}_{{\rm{Alb}}({{\rm{Met}}}^{147}{\rm{O}})}$/${{\rm{C}}}_{{{\rm{Alb}}({\rm{Met}}}^{147})}$ (SD: *p* = 0.0055, r = 0.4592; %CV: *p* = 0.0039, r = 0.4751) (Fig. 4a–c) and the lengths of the hypoglycaemic and hyperglycaemic periods positively correlated with ${{\rm{C}}}_{{\rm{Alb}}({{\rm{Met}}}^{147}{\rm{O}})}$/${{\rm{C}}}_{{{\rm{Alb}}({\rm{Met}}}^{147})}$ (*p* = 0.0402, r = 0.3484 and *p* = 0.0138, r = 0.4124, respectively) (Fig. 4d,f). In contrast, the time spent in the normoglycaemic range negatively correlated with ${{\rm{C}}}_{{\rm{Alb}}({{\rm{Met}}}^{147}{\rm{O}})}$/${{\rm{C}}}_{{{\rm{Alb}}({\rm{Met}}}^{147})}$ (*p* = 0.0026, r = −0.4927) (Fig. 4e).

**Figure 5:**
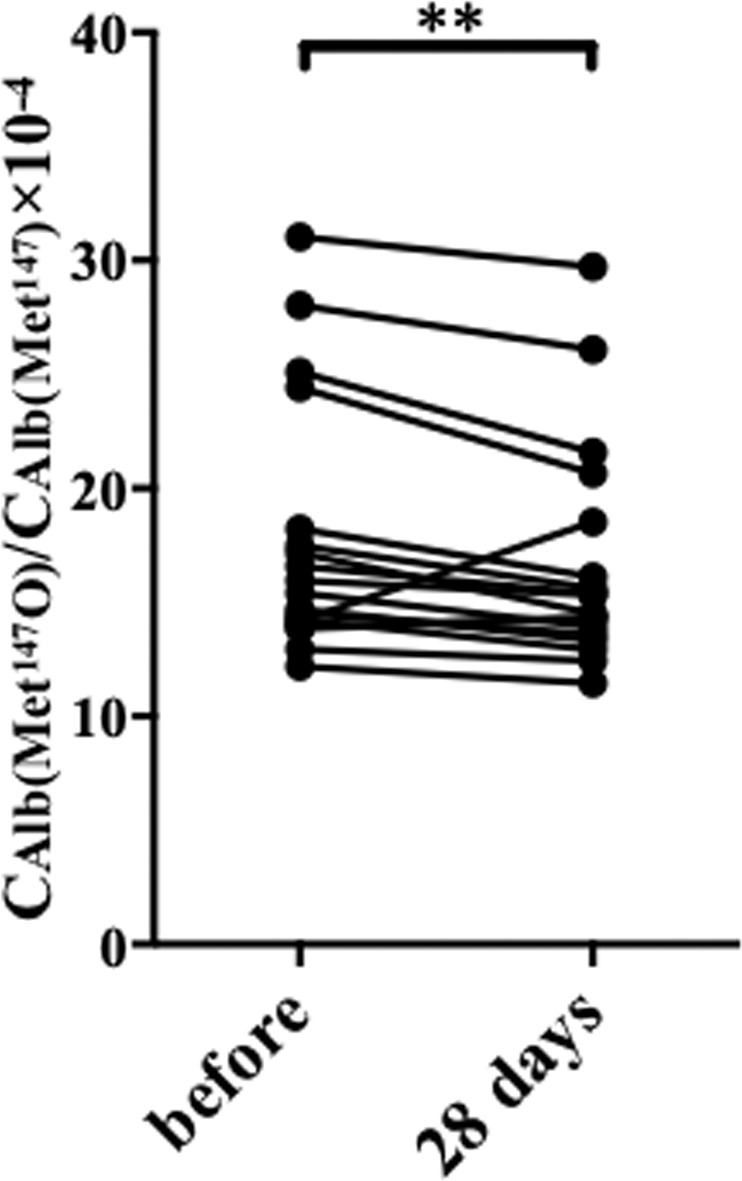
Effect of sodium glucose cotransporter 2 inhibitor treatment on the Met oxidation status of serum albumin. ${{\rm{C}}}_{{\rm{Alb}}({{\rm{Met}}}^{147}{\rm{O}})}$/${{\rm{C}}}_{{{\rm{Alb}}({\rm{Met}}}^{147})}$ was determined in 18 type 2 diabetic participants before and after 28 days’ oral administration of a sodium glucose cotransporter 2 inhibitor. **p < 0.01, calculated using the Wilcoxon signed-rank test.

Because glycaemic excursions appeared to be closely associated with higher ${{\rm{C}}}_{{\rm{Alb}}({{\rm{Met}}}^{147}{\rm{O}})}$/${{\rm{C}}}_{{{\rm{Alb}}({\rm{Met}}}^{147})}$, we next determined whether the administration of a sodium glucose cotransporter 2 inhibitor, which suppresses glycaemic fluctuations, would reduce the oxidation of the Met residue. Indeed, the ${{\rm{C}}}_{{\rm{Alb}}({{\rm{Met}}}^{147}{\rm{O}})}$/${{\rm{C}}}_{{{\rm{Alb}}({\rm{Met}}}^{147})}$ of 18 diabetic participants (nine men and nine women; 54.3 ± 9.5 years; HbA_1c_ 9.3 ± 1.7%, BMI 32.1 ± 5.6 kg/m^2^) was significantly lower after either canagliflozin, luseogliflozin or empagliflozin was administered for 28 days (Fig. 5).

Finally, we assessed the advantage of this updated methodology over our previous method that simply measured signal intensity ratios of Alb(Met^147^O) and Alb(Met^147^) ([Alb(Met^147^O]/[Alb(Met^147^)]) without the use of stable isotope-labelled peptides^28^. We reanalysed all clinical samples pretreated with L-Met and L-Cys to determine [Alb(Met^147^O]/[Alb(Met^147^)] and performed exactly the same statistical analyses as described above. [Alb(Met^147^O]/[Alb(Met^147^)] were significantly higher in the diabetic patients than in healthy volunteers (Supplementary Fig. S2). Unlike ${{\rm{C}}}_{{\rm{Alb}}({{\rm{Met}}}^{147}{\rm{O}})}$/${{\rm{C}}}_{{{\rm{Alb}}({\rm{Met}}}^{147})}$, however, single regression analysis did not reveal the association of [Alb(Met^147^O]/[Alb(Met^147^)] with GA/HbA_1c_ or total bilirubin (Supplementary Table S1). Multiple regression did not identify total bilirubin as an independent variable influencing [Alb(Met^147^O]/[Alb(Met^147^)] (Supplementary Table S2). In patients undergoing continuous glucose monitoring, [Alb(Met^147^O]/[Alb(Met^147^)] only showed a positive correlation with %CV of glucose levels and length of time spent with hypoglycaemia, but did not reveal any significant correlation with standard deviation or average glucose concentration, nor the percentage of time spent with normal or high glucose levels (Supplementary Fig. S2). [Alb(Met^147^O]/[Alb(Met^147^)] of 18 diabetic participants did not significantly decrease after the oral intake of a sodium glucose cotransporter 2 inhibitor for 28 days (Supplementary Fig. S3). These results demonstrate that the updated quantification approach using stable isotope-labelled peptides is far more sensitive in detecting redox status changes in disease pathophysiology over our previous techniques which only measured signal intensity ratios even when excess L-Met and L-Cys were used upon enzyme digestion of serum.

## Discussion

In our previously published study, we determined whether the oxidation of Met residues of serum proteins could be used as a clinical marker of oxidative stress, and concluded that the mass spectral intensity ratio of the levels of oxidised and non-oxidised Met residues of some serum tryptic proteins could be quantified stably and reproducibly^28^. In the present study, we have successfully improved the accuracy and reproducibility of our method for the quantification of the ratio of Met^147^ to Met^147^O-containing tryptic peptides.

First, we measured the concentrations of serum Alb(Met^147^) and Alb(Met^147^O) using stable isotope-labelled peptides by extrapolating the respective XIC data obtained using LC-MS analysis. In our previous method, the mass spectral signal intensity ratio of the serum tryptic peptides with and without MetO was determined^28^. However, this method was limited in accuracy because of broadening of the elution peak, which likely occurs as the LC column condition deteriorates. The degree of this broadening may differ between oxidised and non-oxidised Met, which could lead to lower accuracy when many samples are analysed. The quantification of peptides containing both oxidised and non-oxidised Met that we used in the present study eliminates this drawback by reducing the effects of column condition on the analysis.

Second, the addition of excess L-Met and L-Cys to serum samples prior to the determination of ${{\rm{C}}}_{{\rm{Alb}}({{\rm{Met}}}^{147}{\rm{O}})}$/${{\rm{C}}}_{{{\rm{Alb}}({\rm{Met}}}^{147})}$ also improved the accuracy of LS-MS quantification. Met is prone to spontaneous oxidation once the surfactant used for digesting the serum proteins is removed prior to LC-MS analysis. The Alb(Met^147^) in the trypsinised serum samples becomes significantly oxidised from 1 week of storage, but the addition of excess L-Met prior to this prevents the oxidation of Alb(Met^147^) for at least 1 month. Another difficulty associated with the use of LC-MS technology for the accurate quantification of serum tryptic peptides containing Cys residues is the undesirable carbamidomethylation of peptide N-termini that is associated with the reductive alkylation procedure that prevents disulfide formation. In the present study, we have shown that the addition of excess L-Cys greatly enhances the XIC intensity of non-carbamidomethylated Alb(Met^147^) and SI-Alb(Met^147^). Furthermore, the addition of excess L-Cys and L-Met did not significantly affect ${{\rm{C}}}_{{\rm{Alb}}({{\rm{Met}}}^{147}{\rm{O}})}$/${{\rm{C}}}_{{{\rm{Alb}}({\rm{Met}}}^{147})}$ levels, even after serum samples were stored for 2 years at −80 °C. Thus, the addition of excess L-Met and L-Cys eliminates undesirable spontaneous Met oxidation and carbamidomethylation of N-terminal residues of target peptides, which leads to a marked improvement in the accuracy of the quantification of Alb(Met^147^), Alb(Met^147^O), SI-Alb(Met^147^) and SI-Alb(Met^147^O) using LC-MS.

Having successfully established an accurate, stable and reproducible method for the quantification of the oxidation rate of Met residues in blood proteins using a single drop of human serum, we then determined whether this would reflect the redox status associated with conditions that are known to modulate oxidative stress status in experimental diabetes^30–33^. In humans, it remains controversial whether glucose fluctuation during diabetes activates oxidative stress^34–36^ and it has yet to be established whether hypoglycaemia affects redox status^37^. In the current study, we found that ${{\rm{C}}}_{{\rm{Alb}}({{\rm{Met}}}^{147}{\rm{O}})}$/${{\rm{C}}}_{{{\rm{Alb}}({\rm{Met}}}^{147})}$ was significantly higher in diabetic than in healthy participants. It did not correlate with HbA_1c_ or GA, which reflect mean blood glucose over a period, but significantly correlated with GA/HbA_1c_ ratio, which reflects glycaemic variability^38^. Therefore, we used CGM to determine whether ${{\rm{C}}}_{{\rm{Alb}}({{\rm{Met}}}^{147}{\rm{O}})}$/${{\rm{C}}}_{{{\rm{Alb}}({\rm{Met}}}^{147})}$ reflects features of the pathophysiology of diabetes, such as glucose fluctuations and hypoglycaemia. ${{\rm{C}}}_{{\rm{Alb}}({{\rm{Met}}}^{147}{\rm{O}})}$/${{\rm{C}}}_{{{\rm{Alb}}({\rm{Met}}}^{147})}$ positively correlated with parameters that directly reflect blood glucose fluctuations, such as SD, %CV, duration of hypoglycaemia and duration of hyperglycaemia, and negatively correlated with the length of time the patients were normoglycaemic. In agreement with this, 4 weeks of treatment of 18 type 2 diabetes patients with a sodium glucose cotransporter 2 inhibitor, which reduces glycaemic fluctuation by enhancing urinary glucose excretion, significantly reduced ${{\rm{C}}}_{{\rm{Alb}}({{\rm{Met}}}^{147}{\rm{O}})}$/${{\rm{C}}}_{{{\rm{Alb}}({\rm{Met}}}^{147})}$. Least square multiple regression analysis also showed that serum bilirubin, an endogenous antioxidant^39,40^, HDL-cholesterol and eGFR are independent variables that negatively influence ${{\rm{C}}}_{{\rm{Alb}}({{\rm{Met}}}^{147}{\rm{O}})}$/${{\rm{C}}}_{{{\rm{Alb}}({\rm{Met}}}^{147})}$. Taken together, these findings suggest that a variety of pathophysiological factors that have been shown experimentally to affect oxidative stress, such as glucose fluctuation and hypoglycaemia in diabetes, serum bilirubin, and renal function, do indeed affect indicators of oxidative stress in human blood proteins in a clinical setting.

To assess the superiority of our updated methodology, we reanalyzed all clinical samples using previous methodology that measures signal intensity ratios of MetO- and Met-containing peptides, and compared the results with those of the new method. [Alb(Met^147^O]/[Alb(Met^147^)] levels were not associated with the GA/HbA_1c_ ratio or serum total bilirubin when regression analyses were performed using entire samples. In patients undergoing continuous glucose monitoring, [Alb(Met^147^O]/[Alb(Met^147^)] only showed a positive correlation with %CV of glucose levels and length of time spent with hypoglycaemia, but did not reveal any significant correlation with the standard deviation or average glucose concentration, nor the percentage of time spent with normal or high glucose levels (new Supplementary Fig. S2). In contrast, ${{\rm{C}}}_{{\rm{Alb}}({{\rm{Met}}}^{147}{\rm{O}})}$/${{\rm{C}}}_{{{\rm{Alb}}({\rm{Met}}}^{147})}$ were associated with all of the measured parameters reflecting glycemic excursion, hypoglycaemia, and hyperglycaemia (Fig. 4), and endogenous factors theoretically affecting oxidative stress status, such as serum bilirubin, HDL cholesterol, and eGFR (Table 3). These results demonstrate that the updated approach is far more sensitive in detecting redox status changes over our previous techniques which simply measured signal intensity ratios. To date, clinical biomarkers that can detect the intravascular oxidative stress status elicited by blood glucose fluctuations, hypoglycaemia, and hyperglycaemia in diabetes, as well as that affected by endogenous factors such as serum bilirubin and HDL cholesterol, have been unavailable. Our results demonstrate that our upgraded mass spectrometry approach shows a marked improvement in usefulness such that it can detect the intravascular redox status in human disease pathophysiology.

In our previous report, we identified 53 trypsin-digested serum peptides that contained MetO^28^. However, the XICs of many of their MS1 overlapped with other enzyme-digested peptides when measured by LC-MS with a short analysis time (<60 min), which prevented detection of their signal intensities. MetO-containing peptides derived from low serum concentration proteins elicit comparatively larger background signals, rendering accurate measurement difficult. As a consequence, we selected five methionine-containing peptides technically quantifiable with relatively high concentrations and little signal overlap^28^. Met residues already highly oxidised at baseline in healthy subjects, such as Met^1181^ of complement C3, cannot reflect redox status changes elicited by disease pathophysiology^28^. Therefore, in the current study, we focused on Met^147^ of serum albumin as a promising clinical biomarker candidate because Met^147^ has lower baseline oxidation levels than most other Met residues, such as Met^332^ or Met^363^. The oxidation of two Met residues in the rat brain, Met^288^ and Met^572^, was also reported^41^. Interestingly, these Met residues with elevated baseline oxidation levels are located on the surface of rat and human serum albumin molecules. It is speculated that baseline oxidation and/or susceptibility to oxidation depends upon the position of each Met residue in the three-dimensional structure of the protein molecule. Met is highly susceptible to oxidation, and elevated Met(O)-protein levels have been demonstrated in a variety of oxidative stress-related diseases. Further, Met(O) reductase has not been detected in human blood samples^29^. Therefore, Met(O) in selected Met residues could be used as oxidative stress biomarkers as long as Met- and Met(O)-containing peptides are accurately quantified.

In conclusion, our LC/MS methodology appears to be sufficiently accurate and sensitive for the detection of the effects of intravascular redox status in human pathophysiology.

## Methods

### Participants

The study population consisted of 40 healthy volunteers and 124 diabetic patients (94 with type 2 diabetes and 30 with type 1 diabetes) who visited the Kitasato University Hospital between May 2016 and July 2019. A diagnosis of type 2 diabetes was made on the basis of insulin-independence, according to the criteria of the Japan Diabetes Society (patients with anti-glutamic acid decarboxylase autoantibody >1.5 U/ml or serum C-peptide <0.5 ng/ml were excluded)^42^. Clinical records were reviewed for all the potential participants, and those with acute inflammatory diseases, malignancies, or recent episodes of cerebrovascular or cardiovascular accident were excluded from the study.

### Serum sample collection

Venous blood was collected into vacutainers containing pro-coagulant, allowed to clot at room temperature for approximately 1 h, unless otherwise indicated, and then centrifuged at 1,000 × *g* for 20 min at room temperature. The separated serum was stored at −80 °C until processing. Patients underwent routine evaluations for the systemic diseases covered by the universal health coverage system in Japan^42,43^ using electrocardiography (ECG), radiography (chest and abdomen), ultrasonography (neck and/or abdomen), urinalysis, complete blood count, and the measurement of 15 serum biochemical analysis items. The diabetic participants also underwent ophthalmological and neurological testing, the anti-glutamic acid decarboxylase antibody test, and measurements of GA and/or HbA_1c_, fasting serum insulin, and urinary albumin-to-creatinine ratio.

### Synthesis of stable isotope-labelled peptides

The following two stable isotope-labelled peptides were synthesized by Scrum Inc. (Tokyo, Japan) using L-phenylalanine-N-9-fluorenylmethoxycarbonyl (^13^C_9_, 98%; ^15^N, 98%): SI-Alb(Met^147^), LVRPEVDVMC(Carbamidomethyl)TAFHDNEETFLK, and SI-Alb(Met^147^O), LVRPEVDVM(Oxidation)C(Carbamidomethyl)TAFHDNEETFLK, with the underlined amino acids containing the stable isotope.

### Trypsin digestion of serum proteins

Trypsin digestion of serum proteins was performed essentially as described^44^, but with the following minor modifications. One-hundred-and-ninety-five microlitres of 200 mM triethylammonium bicarbonate, 12 mM sodium deoxycholate, and 12 mM sodium lauryl sulfate was added to 5 μL of thawed serum, and 20 μL of this solution was added to 10 μL of 2.425 μmol/L SI-Alb(Met^147^) or 0.156 μmol/L SI-Alb(Met^147^O), the mixture was vortexed, and then it was mixed with 0.8 μL of 500 mM Bond-Breaker TCEP^TM^ Solution (Thermo Fisher Scientific, MA, USA) and 1.2 μL of 200 mM tetraethylammonium tetrahydroborate (Sigma-Aldrich) and incubated at 50 °C for 30 min. The mixture was then incubated in a dark room at room temperature with 2 μl of 375 mM iodoacetamide (Nacalai Tesque, Kyoto, Japan) for 30 min and then with 2 μl of 400 mM L-Cys (Fujifilm Wako Pure Chemical, Japan) for 10 min, after which 2 μl of 100 ng/μL Lys-C and 2 μl of 100 ng/μL trypsin were added and digestion was carried out for 24 h at 37 °C. Eighteen microlitres of the digest was then mixed with 50 μL of 10% acetonitrile (ACN) and 50 μL of 5% trifluoroacetic acid, and the mixture was centrifuged at 19,000 g for 15 min. The supernatant was recovered and 2 μL of 100 mM L-Met (Tokyo Chemical Industry, Tokyo, Japan) was added, prior to LC-MS analysis.

### Quantification of the oxidised and non-oxidised Met residue-containing tryptic peptides using LC-MS

Analysis of the tryptic digests of the serum samples was performed using LC-MS, essentially as described^28^. Tryptic digests of the serum samples were injected onto a 2.0 mm (inner diameter) × 50 mm CAPCELL PACK MGIII-H S3 column attached to a Nanospace SI-2 HPLC system (Shiseido Fine Chemicals, Tokyo, Japan). The column temperature was maintained at 45 °C. The flow rate of the mobile phase was 200 μL/min, and mobile phase A consisted of 0.05% formic acid (FA) and mobile phase B consisted of 0.05% FA/90% ACN. The mobile phase gradient was programmed as follows: 0% B (0–3 min), 0–55.5% B (3–40 min), 55.5–80% B (40–40.1 min) and 80% B (40.1–45 min). Peptides were introduced from the chromatography column either to an LTQ-Orbitrap Discoverer (Thermo Fisher Scientific) or Q-Exactive (Thermo Fisher Scientific). Full-scan MS spectra were acquired using the Orbitrap (m/z 300–2,000) of an LTQ-Orbitrap Discoverer at a mass resolution of 30,000 at an m/z of 400, while full-scan MS spectra were acquired using the Orbitrap (m/z 300–1,200) of a Q-Exactive at a mass resolution of 70,000 at an m/z of 200. The areas of the XIC for each peptide, Alb(Met^147^O) (*m/z* = 667.3202, *z* = 4), SI-Alb(Met^147^O) (*m/z* = 672.3338, *z* = 4), Alb(Met^147^) (*m/z* = 663.3215, *z* = 4) and SI-Alb(Met^147^) (*m/z* = 665.8283, *z* = 4) were defined following XIC analysis of the LC-MS data using Skyline 3.7.0.11317^45^. ${{\rm{C}}}_{{{\rm{Alb}}({\rm{Met}}}^{147})}$ was extrapolated from the XICs generated from Alb(Met^147^) and SI-Alb(Met^147^), and ${{\rm{C}}}_{{\rm{Alb}}({{\rm{Met}}}^{147}{\rm{O}})}$ from Alb(Met^147^O) and SI-Alb(Met^147^O). The serum ${{\rm{C}}}_{{\rm{Alb}}({{\rm{Met}}}^{147}{\rm{O}})}$/${{\rm{C}}}_{{{\rm{Alb}}({\rm{Met}}}^{147})}$ data obtained using the LTQ-Orbitrap Discoverer and Q-Exactive were strongly correlated (p < 0.0001. r = 0.937, n = 13) and were corrected using the following equation when required:

$$({{\rm{C}}}_{{{\rm{Alb}}({\rm{Met}}}^{147})}/{{\rm{C}}}_{{{\rm{Alb}}({\rm{Met}}}^{147})})=1.12\times {({{\rm{C}}}_{{{\rm{Alb}}({\rm{Met}}}^{147})}/{{\rm{C}}}_{{{\rm{Alb}}({\rm{Met}}}^{147})})}_{{\rm{QE}}}-0.010941$$

### Continuous glucose monitoring

Seven non-diabetic volunteers and 28 diabetic patients underwent continuous glucose monitoring (CGM) using the iPro^TM^ 2 CGM system (Medtronic Minimed Inc. Northridge, CA)^46,47^ for 4–7 days and provided an overnight-fasted blood sample during the period. The complete SGL data were used to assess each glucose profile. The SD and %CV were used to assess glycaemic fluctuations, and the maximum, minimum and mean SGL were used to evaluate glycaemic control. The amount of time over a 24-h period each participant spent in the hypoglycaemic range (SGL < 70 mg/dL), in the normoglycaemic range (70 < SGL < 140 mg/dL) and in the hyperglycaemic range (SGL > 140 mg/dL) were used to analyse their relationships with ${{\rm{C}}}_{{\rm{Alb}}({{\rm{Met}}}^{147}{\rm{O}})}$/${{\rm{C}}}_{{{\rm{Alb}}({\rm{Met}}}^{147})}$ levels.

### Statistical analysis

Data are expressed as mean ± standard deviation unless stated otherwise. Differences in ${{\rm{C}}}_{{\rm{Alb}}({{\rm{Met}}}^{147}{\rm{O}})}$/${{\rm{C}}}_{{{\rm{Alb}}({\rm{Met}}}^{147})}$ levels according to clotting time were analysed using one-way ANOVA. The Mann-Whitney U test was used to compare ${{\rm{C}}}_{{\rm{Alb}}({{\rm{Met}}}^{147}{\rm{O}})}$/${{\rm{C}}}_{{{\rm{Alb}}({\rm{Met}}}^{147})}$ values between diabetic and non-diabetic groups. The remaining comparisons, evaluating the effects of treatment on ${{\rm{C}}}_{{\rm{Alb}}({{\rm{Met}}}^{147}{\rm{O}})}$/${{\rm{C}}}_{{{\rm{Alb}}({\rm{Met}}}^{147})}$ level, were performed using the Wilcoxon signed-rank test for paired data. Linear regression models were used to compare the measured values of ${{\rm{C}}}_{{\rm{Alb}}({{\rm{Met}}}^{147}{\rm{O}})}$/${{\rm{C}}}_{{{\rm{Alb}}({\rm{Met}}}^{147})}$ and to determine the correlations with age, sex, BMI, GA, HbA_1c_ GA/HbA_1c_ and biochemical parameters. Multivariate analyses were performed essentially as described^46,47^, except that age, sex, BMI, GA/HbA_1c,_ eGFR, uric acid, total bilirubin, HDL-cholesterol and the use of metformin, a statin or an angiotensin-converting enzyme inhibitor/angiotensin II receptor blocker were used as explanatory variables, and ${{\rm{C}}}_{{\rm{Alb}}({{\rm{Met}}}^{147}{\rm{O}})}$/${{\rm{C}}}_{{{\rm{Alb}}({\rm{Met}}}^{147})}$ was used as the objective variable. All analyses were performed using GraphPad Prism 5.02 software (GraphPad Software Inc. San Diego, CA, USA) and/or JMP ver. 5.0.1a (SAS, Cary, NC, USA). *P* < 0.05 was considered to represent statistical significance.

### Ethics approval and consent to participate

The protocol was approved by the Kitasato University Medical School/Hospital Ethics Committee (B17-040, B15-181) and written informed consent was obtained from all participants.

All study protocols were performed in accordance with the relevant guidelines and regulations of Kitasato University Medical School as well as the *Ethical Guidelines for Medical and Health Research Involving Human Subjects in Japan* and under the Code of Ethics of the Helsinki Declaration.

## Supplementary information

Supplementary information.

## Data Availability

All data generated or analyzed during this study are included in this article.
