# Supplementary material for: Oxidised Met147 of human serum albumin is a biomarker of oxidative stress, reflecting glycaemic fluctuations and hypoglycaemia in diabetes
Source: Sci Rep. 2020 Jan 14;10:268. doi: 10.1038/s41598-019-57095-2 (PMC6959251; doi:10.1038/s41598-019-57095-2)
Supplement: Supplementary file 1 — Supplementary information. [file 41598_2019_57095_MOESM1_ESM.docx]

**Supplementary Information**

**Oxidised Met^147^ of human serum albumin is a biomarker of oxidative stress, reflecting glycaemic fluctuations and hypoglycaemia in diabetes**

Akari Momozono^1,2,3^, Yoshio Kodera^2,3^, Sayaka Sasaki^1,2,3^, Yuzuru Nakagawa^2^,

Ryo Konno^2^, Masayoshi Shichiri^1*^

^1^Department of Endocrinology, Diabetes and Metabolism, Kitasato University School of Medicine, 1-15-1 Kitasato, Minami-ku, Sagamihara, Kanagawa 252-0374, Japan

^2^Department of Physics, and ^3^Center for Disease Proteomics, Kitasato University School of Science, 1-15-1 Kitasato, Minami-ku, Sagamihara, Kanagawa 252-0373, Japan

Correspondence and requests for materials should be addressed to M.S. (shichiri@kitasato-u.ac.jp)

Supplementary Table S1 **|** Correlations between serum methionine oxidation

determined using signal intensity ratios and other parameters (univariate analyses)

| Parameters | r | p |
| --- | --- | --- |
| Age (years) | 0.1700 | 0.0295 |
| Male: Female |  | 0.7454 |
| BMI | –0.1404 | 0.1005 |
| HbA1c (%) | 0.0869 | 0.3090 |
| GA (%) | –0.0179 | 0.8562 |
| GA/A1c ratio | 0.1893 | 0.0531 |
| BUN (mg/dL) | 0.3444 | <0.0001 |
| Cr (mg/dL) | 0.4354 | <0.0001 |
| eGFR (mL/min/1.73m^2^) | –0.4289 | <0.0001 |
| Uric acid (mg/dL) | 0.1637 | 0.0411 |
| Total bilirubin (mg/dL) | –0.10024 | 0.2192 |
| Triglyceride (mg/dL) | –0.0656 | 0.4099 |
| HDL-cholesterol (mg/dL) | –0.2120 | 0.0073 |
| LDL-cholesterol (mg/dL) | –0.0948 | 0.2347 |

BMI = body mass index; HbA_1c_ = glycated hemoglobin; GA = glycated albumin;

eGFR = estimated glomerular filtration rate; HDL = high-density lipoprotein;

ACEI = angiotensin converting enzyme inhibitor; ARB = angiotensin receptor blocker

Supplementary Table S2 **|** Multivariate analysis of the relationship between serum methionine oxidation determined using signal intensity ratios and other participant characteristics

| Parameters | β | F | p |
| --- | --- | --- | --- |
| Age | –0.1132 | 0.5826 | 0.4483 |
| Male: Female | –0.0197 | 0.0362 | 0.8497 |
| BMI | –0.0478 | 0.0906 | 0.7645 |
| GA/A1c ratio | 0.4704 | 11.9429 | 0.0010 |
| eGFR (mL/min/1.73m^2^) | –0.3962 | 10.2576 | 0.0022 |
| Uric acid (mg/dL) | 0.0993 | 0.4489 | 0.5055 |
| Total bilirubin (mg/dL) | –0.0973 | 0.8793 | 0.3522 |
| HDL-cholesterol (mg/dL) | –0.2742 | 6.1047 | 0.0164 |
| Metformin (+/–) | 0.0929 | 0.6899 | 0.4095 |
| Statin (+/–) | –0.2588 | 5.9857 | 0.0174 |
| ACEI/ARB (+/–) | –0.1644 | 2.1750 | 0.1456 |

BMI = body mass index; HbA_1c_ = glycated hemoglobin; GA = glycated albumin;

eGFR = estimated glomerular filtration rate; HDL = high-density lipoprotein;

ACEI = angiotensin converting enzyme inhibitor; ARB = angiotensin receptor blocker


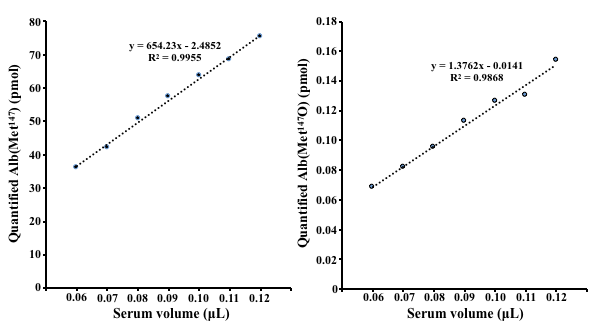


**Supplementary Figure S1** **| Calibration curves for the absolute quantification analyses of serum Alb(Met^147^) and Alb(Met^147^O) using stable isotope-labeled peptides**. Indicated volumes of serum were pre-mixed with SI-Alb(Met^147^) (4.85 pmol) and SI-Alb(Met^147^O) (0.312 pmol), digested with trypsin and analysed by LC-MS. XIC intensities of Alb(Met^147^), Alb(Met^147^O), SI-Alb(Met^147^) and SI-Alb(Met^147^O) were determined, and the absolute amounts of endogenous Alb(Met^147^) and Alb(Met^147^O) in respective diluted serum samples were extrapolated from XICs generated by the relevant peptides and the amount of spiked stable isotope-labelled peptides. The resulting equations from linear regression analysis for the amount (pmol) of Alb(Met^147^) and Alb(Met^147^O) versus serum volume were y = 654.23 × –2.4852 (R^2^=0.9955) and y = 1.3762 × – 0.01411 (R^2^=0.9868), respectively.


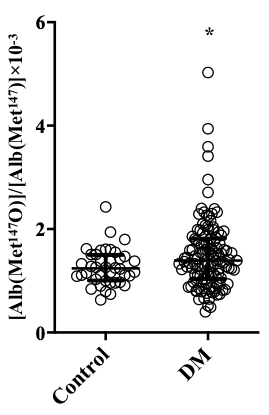


**Supplementary Figure S2** **| Levels of Met oxidation in diabetic and non-diabetic participants as determined by signal intensity ratios of Alb(Met^147^) and Alb(Met^147^O) without the use of stable isotope-labelled peptides.** The signal intensity ratios of Alb(Met^147^O) and Alb(Met^147^), [Alb(Met^147^O)]/[Alb(Met^147^)], were determined by LC-MS analyses of 40 healthy volunteers (Control) and 124 diabetic participants (DM). *, *p*<0.05 compared with Control.

**
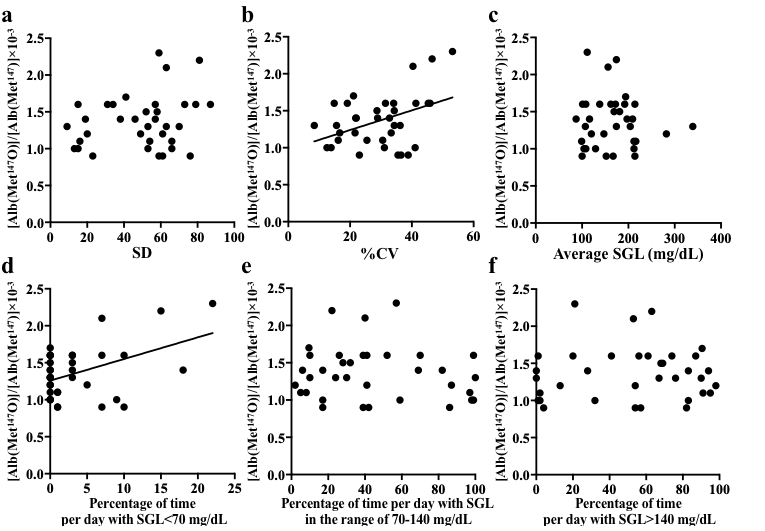
**

**Supplementary Figure S3** **|** **Relationship between Met oxidation as determined by signal intensity ratios and blood glucose profile, evaluated using continuous glucose monitoring.** Continuous glucose monitoring was performed in 35 participants for 4–7 days, and sensor glucose levels (SGL) over the entire monitoring period were used to calculate the standard deviation (SD) (**a**), % coefficient of variation (%CV) (**b),** and average SGL (**c**) values. The relative lengths of time with SGL <70 mg/dL (**d**), 70–140 mg/dL (**e**), and >140 mg/dL (**f**) were plotted against [Alb(Met^147^O)]/[Alb(Met^147^)] values, and the corresponding regression line with significant correlation is shown.


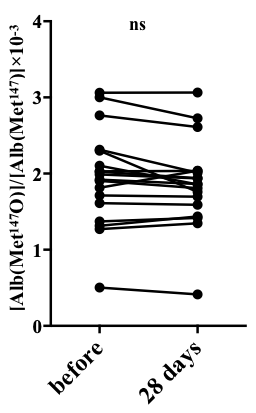


**Supplementary Figure S4** **| Effect of sodium glucose cotransporter 2 inhibitor treatment on the Met oxidation status as determined by signal intensity ratios of Alb(Met^147^) and Alb(Met^147^O).** [Alb(Met^147^O)]/[Alb(Met^147^)] was determined in 18 type 2 diabetic participants before and after 28 days of oral administration of a sodium glucose cotransporter 2 inhibitor. ns, *not significant*.
